# Recent Advances in Microswimmers for Biomedical Applications

**DOI:** 10.3390/mi11121048

**Published:** 2020-11-27

**Authors:** Ada-Ioana Bunea, Rafael Taboryski

**Affiliations:** National Centre for Nano Fabrication and Characterization (DTU Nanolab), Technical University of Denmark, Ørsted Plads 347, 2800 Lyngby, Denmark; rata@dtu.dk

**Keywords:** microswimmer, microrobot, biomedical application, drug delivery, sensing, imaging

## Abstract

Microswimmers are a rapidly developing research area attracting enormous attention because of their many potential applications with high societal value. A particularly promising target for cleverly engineered microswimmers is the field of biomedical applications, where many interesting examples have already been reported for e.g., cargo transport and drug delivery, artificial insemination, sensing, indirect manipulation of cells and other microscopic objects, imaging, and microsurgery. Pioneered only two decades ago, research studies on the use of microswimmers in biomedical applications are currently progressing at an incredibly fast pace. Given the recent nature of the research, there are currently no clinically approved microswimmer uses, and it is likely that several years will yet pass before any clinical uses can become a reality. Nevertheless, current research is laying the foundation for clinical translation, as more and more studies explore various strategies for developing biocompatible and biodegradable microswimmers fueled by in vivo*-*friendly means. The aim of this review is to provide a summary of the reported biomedical applications of microswimmers, with focus on the most recent advances. Finally, the main considerations and challenges for clinical translation and commercialization are discussed.

## 1. Introduction

Microswimmers, which are microscopic objects with the ability to move in liquid environments, were pioneered in the beginning of the third millennium. Although only two decades have passed since their emergence, microswimmers have already shown great promise for various biomedical and environmental applications. Given the recent nature of the field, there is yet no consensus in the literature for the nomenclature of the microscopic objects this article refers to as “microswimmers”. Among the many alternative names such objects are given in the literature, microswimmers, micro/nanorobots and micro/nanomotors are likely the most frequently encountered. Other common terms may be more descriptive, including information about the object shape, e.g., microtube or microhelix, its components, e.g., biohybrid, spermbot [1], bacteriabot [2], or micro-bio-robot [3], or behavior, e.g., microrocket, microbullet, microtool or microroller. Researchers have also named their microswimmers e.g., medibots [4], hairbots [5], iMushbots [6], IRONSperm [7], teabots [8], biobots [9], T-budbots [10], or MOFBOTS [11]. In this review, the term “microswimmer” is used for all the aforementioned objects, whereas more specific terms are only employed if they bring additional useful information.

**Figure 1:**
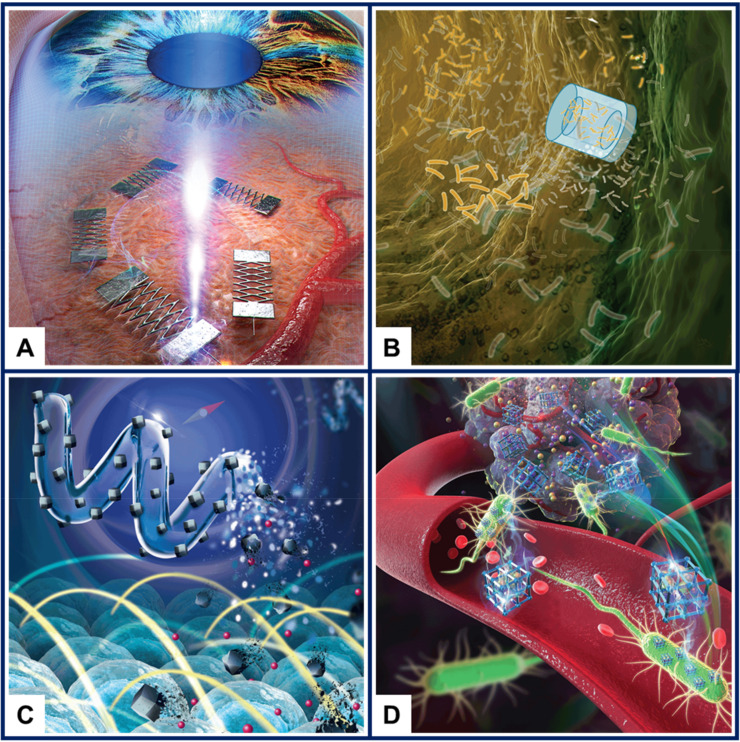
Conceptual drawings showing microswimmers with biomedical applications. (**A**) Laser-controlled Ni/Ti shape memory alloy microswimmer for intraocular surgery. Figure reproduced from the front cover of *Advanced Materials Technologies*, [14], © 2019 WILEY-VCH Verlag GmbH. (**B**) Microcontainers for improved biofilm treatment. Figure reproduced from the front cover of *Advanced Healthcare Materials*, [15], © 2020 WILEY-VCH Verlag GmbH. (**C**) Biodegradable metal-organic framework-based microswimmer for targeted drug delivery. Figure reproduced from the front cover of *Advanced Healthcare Materials*, [16], © 2020 Wiley-VCH GmbH. (**D**) Biohybrid microrobot comprising *Escherichia coli* biomineralized using a metal–organic framework employed for targeted drug delivery. Figure reproduced from the back cover of *Advanced Healthcare Materials*, [17], © 2020 WILEY-VCH Verlag GmbH.

As it is the case for micro- and nanotechnology in general, the history of microswimmers arguably starts with Richard Feynman’s famous speech “There’s plenty of room at the bottom” [12]. In the visionary speech, among other topics, Feynman addressed the idea of microscopic surgeons, saying: “A friend of mine (Albert R. Hibbs) suggests a very interesting possibility for relatively small machines. He says that, although it is a very wild idea, it would be interesting in surgery if you could swallow the surgeon. You put the mechanical surgeon inside the blood vessel and it goes into the heart and <<looks>> around (of course the information has to be fed out). It finds out which valve is the faulty one and takes a little knife and slices it out. Other small machines might be permanently incorporated in the body to assist some inadequately-functioning organ.” The concept of the surgeon one could swallow was soon after presented in the science-fiction movie “Fantastic Voyage” and in Isaac Asimov’s novelization. Only a few decades later, microswimmers aiming to become true microscale surgeons evolved from an intriguing science-fiction concept to a reality explored in many research laboratories around the world, as already highlighted by Sitti in 2009 [13]. Figure 1 shows examples of conceptual drawings of microswimmers with biomedical applications that were featured on the cover of reputed scientific journals in the past two years.

A big part of modern-day research is focused on improving our quality of life. Among other relevant subjects, biomedical studies are a particularly important element contributing to the quality of life, in a world where life expectancy keeps increasing and the need for personalized medicine becomes more and more obvious. Thus, when novel and clever technological solutions, such as microswimmers, are developed, it is natural to explore their potential for biomedical applications. Already in 2010, Nelson et al. reviewed the existing and envisioned applications of microrobots in minimally invasive medicine [18]. Since then, the field has grown tremendously, and it has become obvious that the potential of microswimmers for biomedical applications is outstanding. Already, many interesting tasks can be performed in vitro using tailored microswimmers. Still, a number of challenges regarding e.g., in vivo control, biocompatibility and long-term biosafety need to be overcome before microswimmers can become a viable option for many clinical applications [19].

**Figure 2:**
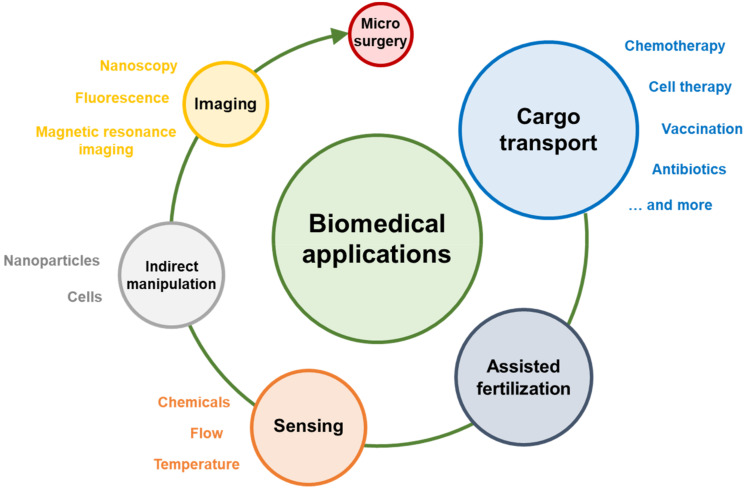
Schematic representation of the biomedical applications of microswimmers. The figure also serves as a guide for the structure of this review.

A schematic representation of the classification of biomedical applications covered in this review is shown in Figure 2, which also serves as a visual guide for the structure of the review. First, the use of microswimmers for cargo transport in drug delivery and other biomedical applications is discussed at length. Subsequently, assisted fertilization, sensing, micromanipulation, imaging and other interesting examples of microswimmer biomedical applications are surveyed. Some of the more complex microswimmers could easily fit into multiple categories, as they are applied simultaneously for e.g., sensing and drug delivery. In this case, the microswimmers were included in the category corresponding to their most novel attributes.

There are many different types of microswimmers, which are powered and actuated in various ways. Many swimming strategies for individual microswimmers [2,20,21,22,23,24] and swarms [25,26,27,28,29,30] were explored throughout the years. Typically, microswimmers either rely on external power sources, as it is the case for e.g., magnetic [31], optic [9], or acoustic [32] control, or employ the fuel available in their surroundings, in the case of biohybrids or catalytic microswimmers. Magnetic and acoustic actuation are typically compatible with in vivo microswimmer manipulation and catalytic microswimmers can be specifically engineered to employ in vivo fuels. The use of optical forces in biological fluids or in vivo is more challenging, but interesting examples have been nevertheless demonstrated [9]. Often, researchers choose to take inspiration from nature, either for the entire microswimmer design, or for achieving a desired propulsion type. For example, one of the first bioinspired microswimmers consisted of human red blood cells modified with a flagellum-like artificial component made of filaments of magnetic particles bonded via biotin–streptavidin interactions [33]. More recently, biomimetic swimming inspired by e.g., worm-like travelling wave features [34], shrimp locomotion [14], and bacterial “run-and-tumble” [35], was demonstrated by using shaped light. A different nature-inspired approach is the use of biohybrid microswimmers comprising a living component and a synthetic one. Biohybrids most often take advantage of the microscale motion of various biological systems and can also make use of other behaviors characterizing the living component [36]. For magnetic bioinspired and biohybrid microswimmers, typical model organisms are bacteria, sperm cells and magnetotactic cells [37]. In addition to the use of magnetic forces, actuation of bioinspired microswimmers was also demonstrated using e.g., acoustic excitation [38] or optical forces [39]. Another interesting nature-inspired behavior related to optical forces is that of phototaxis, which can be exploited by e.g., cargo-carrying microroganisms [40], synthetic microswimmers [41,42,43] or biohybrid microswimmers [44]. For more information on microswimmer actuation, interested readers are directed to review papers which are focused on explaining or comparing the existing propulsion and control strategies [45,46,47,48,49]. Nevertheless, it should be mentioned here that magnetic actuation is most often included for controlled in vivo guiding, even for microswimmers which rely on a different type of propulsion. In a recent review, Koleoso et al. discuss the use of magnetic small scale robots for biomedical applications and provide details about the various magnetic fields and actuation systems developed for such purposes [31].

The fabrication and functionalization of microswimmers is also an interesting and vast area. Briefly, fabrication strategies include e.g., two-photon polymerization (2PP) 3D printing, photolithography, template-assisted electrodeposition, or bonding of a living component to an inanimate one by exploiting different strategies. Approaches that are even more recent exploit 4D printing, which is the 3D printing of stimuli-responsive materials [50,51,52,53]. Further functionalization is often required, either to enable a certain type of actuation, e.g., metal coating for magnetic control or thermoplasmonic responses, or as part of the application, if certain characteristics are required for e.g., sensing, cargo transport, controlled interactions with the environment, or biodegradation [54,55].

Although definitely interesting, the many actuation and fabrication strategies will not be further described as such as part of this review, which is instead focused on microswimmer applications. While some pioneering works, unique examples, or other review papers are mentioned briefly, this paper expands on interesting examples of microswimmers for biomedical applications reported in the last two years.

## 2. Cargo Transport and Drug Delivery

Cargo transport is one of the most explored microswimmer-enabled tasks. In the context of biomedical applications, cargo transport most often implies the delivery of different types of drugs, but it has also been exploited for e.g., cell therapy. By tailoring the propulsion mechanism and other microswimmer characteristics, it is possible to achieve targeted delivery, which is one of the important characteristics of drug delivery systems. Whereas many examples have already been demonstrated in vitro, and several even in vivo in small animals, clinical translation remains a bottleneck. Nevertheless, the use of biodegradable smart materials combined with magnetic control and selective release or targeting strategies seems to hold the most promise for applications in the human body.

Drug delivery systems are typically nanoparticles that need to have the ability to uptake a therapeutic agent and release at the target site. For nanosized drug delivery systems, chemical composition and surface functionalization are most often essential, and the strategies for their functionalization for targeted drug delivery were reviewed elsewhere [56]. While microswimmers can of course take advantage of functionalization, their complex shapes and various control mechanisms allow a more complex tailoring of the drug delivery process compared to standard drug delivery systems with smaller dimensions.

Catalytic microswimmers were used soon after their development for cargo transport. Already in 2008, colloidal cargo was transported by Janus catalytic microswimmers after attachment using either electrostatic interactions or streptavidin coupling [57]. The same year, cargo pick-up and release was demonstrated with the aid of carbon nanotube-based microswimmers with both magnetic and catalytic abilities [58]. Soon after, controlled drop-off of a pre-loaded cargo was demonstrated by applying photochemical stimuli. Subsequently, targeted drug delivery was achieved using microswimmers relying on magnetic actuation. Examples include 2PP 3D printed polymeric helical microswimmers coated with a nickel/titanium bilayer [59], flexible magnetic nickel/silver microswimmers [60], biodegradable gelatin rockets able to release a pre-loaded drug upon near-infrared light illumination due to photothermal effects [61], or nickel/titanium magnetic helical microswimmers for targeted gene delivery [62]. In the following years, cargo transport was reported using bacterial [63,64] and bacteria-driven [2,65] microswimmers.

Erkoc et al. recently reviewed the existing strategies and demonstrations of microswimmers in active therapeutic delivery [66], whereas the current challenges and design considerations for developing microswimmers as targeted drug delivery systems were highlighted by Singh et al. [67] and Agrahari et al. [68]. Self-propelled microswimmers for cargo transport were also reviewed recently [69]. Nevertheless, the last couple of years have brought new and interesting demonstrations of microswimmers involved in cargo transport for different types of biomedical applications. Selected examples are discussed below.

### 2.1. Chemotherapy

**Figure 3:**
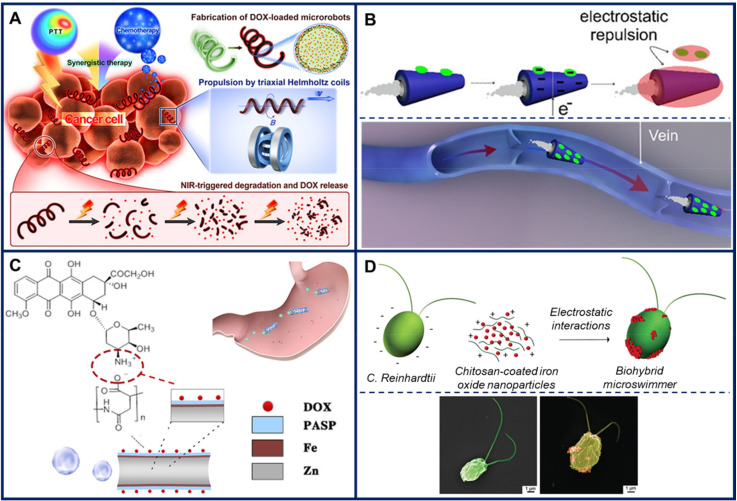
Recent examples of microswimmers developed for the delivery of chemotherapeutic agents. (**A**) *Spirulina-*based magnetic helical microswimmers loaded with doxorubicin (DOX). The microswimmers were shown to fight cancer in vitro by synergistic photothermal effects and DOX release upon near infrared-triggered degradation. Figure reproduced from [71], © 2019 American Chemical Society. (**B**) Top: Mechanism of the electrochemical release of DOX from a bismuth/nickel/platinum tubular microswimmer upon applying a negative potential (−1 V). Drug release is attributed to an increase in the negative repulsive charge in the system. Bottom: Microswimmer navigation inside a vein. Figure reproduced from [75], © 2019 American Chemical Society. (**C**) Microswimmer engineering for in vivo DOX delivery in mouse stomachs. A zinc core is surrounded by a thin iron layer, which is in turn covered by poly(aspartic acid) (PASP) which can bind DOX through electrostatic interactions. In vivo manipulation was based on magnetic control. Figure reproduced from [77], © 2019 American Chemical Society. (**D**) Top: Production steps for biohybrid *Chlamydomonas reinhardtii* microswimmers. Bottom: Pseudocolored scanning electron microscope images of bare microalgae (left) and biohybrid microalgae covered by chitosan-coated iron oxide nanoparticles, shown in orange (right). Figure adapted from [44], © 2020 by the authors (Creative Commons Attribution license).

Chemotherapeutic agents typically have high toxicity, as their main task is to disrupt the normal cell cycle in cells in order to slow down or prevent the proliferation of abnormal cancer cells. This leads to numerous side effects, as the cell cycle of healthy cells is also affected. Consequently, there is a huge interest in developing solutions for targeted cancer therapy [70], which is why many cargo transport demonstrations in microswimmers focus on the delivery of chemotherapeutic agents. Figure 3 shows a few recent examples.

*Spirulina-*based microswimmers were engineered to incorporate core-shell structured palladium/gold nanoparticles, magnetite (Fe_3_O_4_) nanoparticles, and the chemotherapeutic agent doxorubicin [71]. In vitro testing confirmed doxorubicin release upon pH changes or irradiation using near-infrared light, as well as the presence of synergistic photothermal effects induced with the aid of the palladium/gold nanoparticles, as schematically shown in Figure 3A.

Another type of degradable microswimmer with magnetic control was reported by Park et al. [72]. The helical microswimmers were made by 2PP 3D printing of a mixture containing the polymers poly(ethylene glycol) diacrylate (PEGDA) and pentaerythritol triacrylate (PETA), magnetite nanoparticles, and the chemotherapeutic agent 5-fluorouracil. In the in vitro study conducted, in addition to their ability to transport and release the drug at the desired site, the microswimmers were also able to induce a hyperthermic effect in the nearby cell population, which provided a complementary mode to kill the cancer cells and thus treat cancer.

Another example of biocompatible microswimmer employed for drug delivery came from Darmawan et al., who produced a self-folding helical magnetic structure that could rapidly release the pre-loaded drug doxorubicin upon ultrasonic stimulation [73].

Magnetic and pH-sensitive double-layer microswimmers were employed for sustained drug release in vitro [74]. Two different designs were investigated, thumbtack-like and frisbee-like. The microswimmers contained magnetite nanoparticles, as well as doxorubicin, embedded first in a chitosan matrix and then into a calcium alginate hydrogel. The calcium alginate hydrogel shrinks in an acidic environment, protecting the embedded microparticles, but it swells and ultimately dissolves in alkaline conditions, allowing for the sustained release of its cargo.

Bismuth/nickel/platinum tubular microswimmers loaded with doxorubicin were employed for localized release in microfluidic channels and on cancer cell populations based on an electrochemical release mechanism [75]. Briefly, ultrafast drug release from the microswimmers was achieved by performing cyclic voltammetry, as electron injection into the microswimmers caused electrostatic repulsions leading to doxorubicin release (Figure 3B). Subsequently, intracelullar uptake of doxorubicin was followed by the appearance of apoptotic features in the cancer cells.

Microswimmers reported by Lee et al. were designed to include a needle-type feature for piercing through the target microtissue and a scaffold body for increased surface area and drug loading ability [76]. The microswimmers were coated with nickel and titania (TiO_2_) for magnetic actuation, and subsequently loaded with the chemotherapeutic agent paclitaxel. The needle-type feature enabled the microswimmers to spear through the target cell population for fixation, and reduced cancer cell viability was observed for the paclitaxel-loaded microswimmers.

MOFBOTS, or metal-organic framework-based biomedical microrobots, were recently reported [11,16]. In the early stage of the study, helical microswimmers were fabricated by 2PP 3D printing, coated with a thin layer of nickel or titanium for magnetic actuation, and subsequently with the zeolitic imidazole framework-8 [11]. The fluorescent dye rhodamine B was employed as model drug to demonstrate the ability of MOFBOTS to transport cargo. Expanding on this principle, biodegradable MOFBOTS were developed soon after and employed in vitro for doxorubicin delivery [16].

A different type of biodegradable microswimmer, microrockets propelled by gastric acid, was developed by Zhou et al. [77]. The microswimmers consisted of a poly(aspartic acid) microtube surrounding a zinc core covered with a thin iron layer. Drug loading with doxorubicin relied on electrostatic interactions between the poly(aspartic acid) and the drug, transport to the stomach was controlled using an external magnet, and the acidic pH in the stomach triggered bubble propulsion of the microrockets due to the galvanic corrosion of the zinc core (Figure 3C). Doxorubicin release was demonstrated in vivo in mouse stomachs.

Using a similar approach, Liu et al. developed organic–inorganic Janus microswimmers with a magnesium core partially surrounded by a mixture of poly(lactic-*co*-glycolic acid) (PLGA) and doxorubicin [78]. The microswimmers are propelled by hydrogen bubbles produced during the catalytic reaction between the magnesium core and water. The hydrogen’s ability to scavenge reactive oxygen species was shown to synergistically complement the chemotherapeutic agent in vitro.

Silica Janus microspheres with a magnetic gold/nickel coating were employed for targeted doxorubicin delivery [79]. The leukocyte-inspired microswimmers were functionalized with antibodies targeting cancer cells and with light-cleavable doxorubicin. Propulsion in ex vivo mouse blood circulated using a physiologically relevant blood flow was demonstrated. Furthermore, the presence of targeting antibodies enabled specific targeting of cancer cells in vitro, and doxorubicin was released after reaching the target cells by illumination with ultraviolet light.

Biohybrid microswimmers for the delivery of chemotherapeutic agents were reported by Akolpoglu et al. [44]. The living component *Chlamydomonas reinhardtii,* a type of biflagellated unicellular green microalgae, was coated with chitosan by exploiting electrostatic interactions between the positively charged natural polymer and the negatively charged alga wall (Figure 3D). Chitosan further acted as a binding agent, enabling the attachment of magnetic nanoparticles. Thus, the biohybrid microswimmer was amenable to magnetic manipulation, and exhibited the phototactic behavior specific to its green microalga component. For the in vitro drug delivery demonstration, doxorubicin was attached to the iron oxide nanoparticles using a photocleavable linker, and drug release was stimulated by ultraviolet light.

While the two approaches described above are quite interesting for in vitro studies, the delivery of ultraviolet light is rather difficult to achieve in vivo, as well as potentially dangerous to the human body. Nevertheless, replacing the photocleavable linker employed for doxorubicin coupling with a different type of linker should help overcome this.

### 2.2. Cell Therapy

Cell therapy involves the transplantation of healthy cells into a patient, either with the purpose of providing a long-term replacement of cells from damaged tissues, or of releasing soluble therapeutic factors. Localized delivery of the cells is of course highly desirable, which is why microswimmer carriers seem a promising option.

Capsule-type microswimmers with pick-and-drop motion were developed by Lee et al. [80]. The 3D printed microswimmers comprised two components, a plunger and a cap, and were controlled wirelessly by magnetic forces. The capsule-type microswimmers could deliver cells in suspension, adherent cells, or drugs, while at the same time protecting the cargo from shear forces.

Yasa et al. reported 3D printed magnetic microswimmers which included a transport compartment for cell therapy referred to as a recapitulated stem cell niche [81]. Selective patterning enabled functionalization of the transport compartment with collagen I, hyaluronan and fibronectin. Functionalization with these key components of the native extracellular matrix, combined with cell–cell interactions dictated by the material properties and cell loading, resulted in a recapitulated stem cell niche mimetic microenvironment as an integrated component of the cell carrier microswimmers.

**Figure 4:**
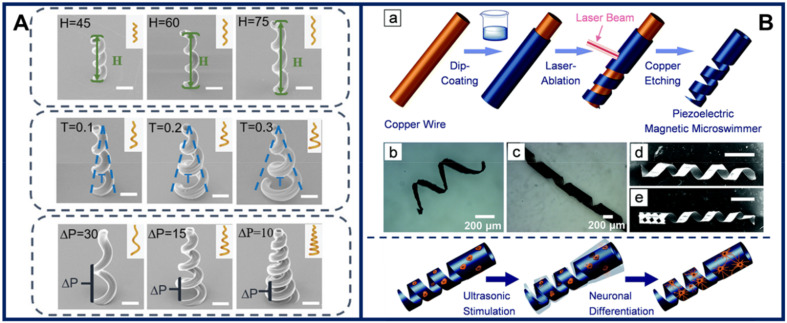
Recent examples of microswimmers developed for cell therapy. (**A**) Scanning electron micrographs of hollow microhelix magnetic microswimmers able to transport nanoparticles and cells. Scale bars, 20 µm. Figure reproduced from [82], © 2019 WILEY-VCH Verlag GmbH & Co. (**B**) Piezoelectric magnetic microswimmers for cell differentiation into neurons and subsequent delivery. Top: (a) fabrication steps. (b,c) Optical images of microswimmers with different design parameters. (d,e) Micrographs of microswimmers with and without a “head”. Bottom: cell culture and differentiation schematics. Figure reproduced from [84], © 2019 by the authors (Creative Commons Attribution license).

Conical hollow magnetic microhelices fabricated by 2PP 3D printing were shown to efficiently transport nanoparticles and cells [82]. As the microswimmers have a hollow-core structure, numerous nanoparticles can be loaded onto them, inside the hollow core, as well as on the surface. Furthermore, coating of the microswimmers with a biocompatible poly-L-lysine layer enabled attachment of neural stem cells, whereas prolonged cell culture ultimately led to cell migration away from the microswimmer, which is highly desirable for cell therapy. Figure 4A shows different examples of conical hollow microhelices. Helical microswimmer cell carriers based on biocompatible polymers were also demonstrated [83].

Piezoelectric magnetic microswimmers for cell differentiation and neuron delivery were developed by Chen et al. [84]. The microswimmers were fabricated by first dip-coating a tubular copper substrate with a mix of CoFe_2_O_4_ magnetic nanoparticles and a piezoelectric polymer solution (polyvinylidene fluoride-*co*-trifluoroethylene or poly-L-lactic acid), followed by laser ablation and copper etching. Prior to cell seeding, the microswimmers were coated with poly-L-lysine. Stem cell differentiation into neuronal cells was achieved by ultrasound stimulation in cell populations attached to the microswimmers, bypassing the need for using differentiation factors. Schematics of the fabrication, and cell culture and differentiation processes are shown in Figure 4B. The microswimmer ability to move in viscous media was assessed in silicon oil. Both actuation mechanisms, i.e., magnetic motion control and ultrasonic differentiation stimulation, can be delivered in vivo, but their long-term biosafety, as well as that of the microswimmer component materials, still needs to be evaluated.

Scaffold-type microswimmers with cylindrical, hexahedral, helical and spherical shapes for stem cell transplantation were developed by Jeon et al. [85]. The scaffold-like structure conferred porosity to the microswimmer and greatly increased the cell loading capability compared to other similar cell carriers. Stem cell culture and differentiation on the scaffolds were demonstrated, as well as in vivo transportation in mice using magnetic actuation.

Soft magnetoelectric microswimmers with the ability to stimulate the differentiation of neuronal stem cells, in addition to their function as cell carriers, were recently reported [86]. The microswimmers were fabricated in gelatin-methacryloyl (GelMA), a hydrogel which can be degraded by proteases released from human cells and loaded with magnetoelectric nanoparticles composed of a CoFe_2_O_4_ core and a BiFeO_3_ shell. These nanoparticles were not only biocompatible, but also able to induce neuronal differentiation, particularly upon magnetic stimulation. The biocompatibility and ability to degrade of the microswimmers shown in vivo in mice highly recommend them for clinical applications, although the long-term biosafety of the magnetoelectric nanoparticles still needs to be investigated.

### 2.3. Vaccination

Oral administration of vaccines in conventional formulations is most often ineffective, which is why most vaccines are instead administered using injections. Various drug delivery systems, including microswimmers, might hold the key for improving the bioavailability of vaccines upon oral administration and thus fostering oral vaccination.

**Figure 5:**
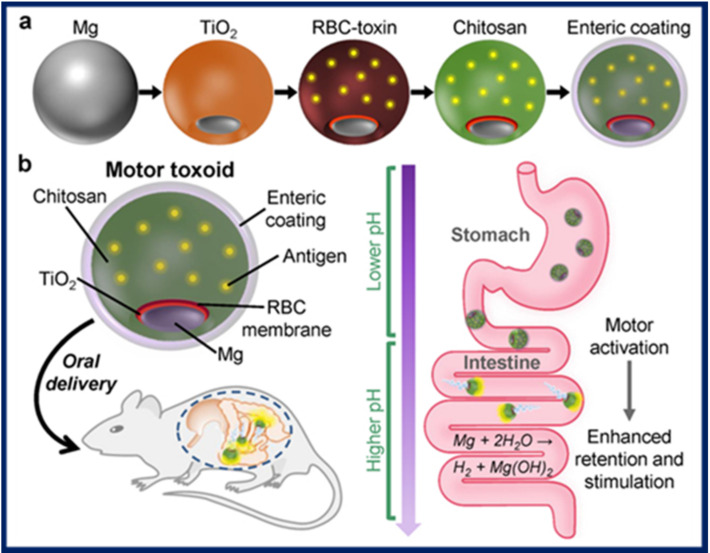
(**a**) Microswimmer fabrication steps: magnesium microparticles are first partially coated with titania, then with a red blood cell (RBC) membrane containing the toxin, chitosan for mucoadhesion, and finally with an enteric coating resistant to acidic pH. (**b**) Principle of action for oral vaccination in mice using the microswimmers (motor toxoids) as delivery agents. Figure reproduced from [87], © 2019 American Chemical Society.

Biomimetic microswimmers were employed as delivery agents for oral vaccination in mice [87]. The microswimmers were fabricated by a sequential process in which magnesium microparticles were asymmetrically coated with titania, followed by coating with a toxin-containing red blood cell membrane as antigenic material, chitosan as mucoadhesive layer, and finally, a pH-sensitive enteric coating to protect the microswimmer from degradation in the acidic stomach environment. Upon oral administration to mice, the microswimmers travel to the intestine, where the coating dissolves due to pH changes and the intestinal fluid activates autonomous propulsion, which facilitates enhanced penetration in the intestinal wall, where the mucoadhesive coating ensures retention so that the antigen release can take place. The schematic representation of the fabrication process and of the microswimmer route in vivo is shown in Figure 5.

### 2.4. Other Types of Cargo

Various types of cargo delivery relevant for biomedicine were reported during the past two years. For example, anion delivery was demonstrated by Beladi-Mousavi et al. using self-propelled microswimmers consisting of an inner platinum catalytic layer, a middle nickel magnetic layer, and an outer polycationic viologen layer [88]. The anion-loaded microswimmers were shown to be stable under ambient conditions for at least four months, and controlled release with high efficiency was demonstrated using either electrochemical, photochemical or a metathesis reaction.

On the other hand, delivery of iron and selenium minerals for the treatment of anemia was demonstrated using magnesium microspheres coated with titania for magnetic actuation and several polymer layers with different functions [89]. The magnesium/titania Janus particles were further coated with poly(lactic-*co*-glycolic acid) to protect them from premature inactivation, then with a chitosan layer containing the minerals, and finally with an enteric coating which ensures microswimmer integrity in the gastrointestinal tract up until the duodenum. Active mineral replenishment was demonstrated in vivo in an anemic mouse model.

Yan et al. reported biohybrid magnetic microswimmers engineered from multicellular *Spirulina platensis* algae [90]. The microswimmers were loaded with human mesenchymal stem cell growth factors using controlled rehydration of the lyophilized algae, their movement upon magnetic actuation was demonstrated in a sinuous microfluidic channel filled with mouse intestinal fluids, and cargo release was based on biodegradation and was noticeable for up to 25 days. The in vivo studies conducted confirmed localized delivery of the growth factors and their bioactivity.

So-called teabots, bioinspired microswimmers comprising unfermented white tea buds from *Camellia sinensis,* known to contain polyphenols with health benefits, and the antioxidant ascorbic acid, were reported by Bhuyan et al. [8]. The teabots were propelled using ultrasound and their effect on amyloid disintegration and reducing in vitro oxidative stress was found to be quite promising. Based on a similar approach, *Camellia sinensis*-based T-budbots containing magnetite nanoparticles were loaded with the antibiotic ciprofloxacin and employed in biofilm antibacterial studies [10].

Another interesting example of drug delivery for biofilm treatment was reported by Birk et al. [15]. SU-8 microcontainers fabricated by photolithography were loaded with the antibiotic ciprofloxacin and coated with a lid consisting of either poly(ethylene glycol), chitosan or Eudragit S100. The antibiotic effect was demonstrated in vitro on *Pseudomonas aeruginosa* biofilms. Although the microcontainers do not support external motion control and therefore do not fall into one of the typical microswimmer categories, they are rather large and embody a clever engineering solution for drug delivery, which is why they were included in the review.

The latest trend involves using the microswimmers themselves as “cargo”, in a similar approach to that common to smaller drug delivery systems. Hortelão et al. developed urease-powered microswimmers based on mesoporous silica nanoparticles functionalized on their outer surface with both poly(ethylene glycol) and an antibody targeting bladder cancer cells. Bladder cancer spheroids internalized the microswimmers, leading to their disintegration and cell death. In addition to its role in targeting and internalization, the antibody also leads to reduced proliferation in the cancer cells. A recent review from Venugopalan et al. focused on the existing demonstrations and future prospects of microswimmers for intracellular applications [91].

## 3. Assisted Fertilization, Sensing, Indirect Manipulation, Imaging, and Microsurgery

### 3.1. Spermbots and Assisted Fertilization

In recent years, the demand for assisted reproductive technology has increased, as the technology matured and became more effective and affordable. Assisted reproductive technology is meant to help bypass the inability to conceive naturally, which has multiple causes in both men and women. In vitro fertilization, followed by the implantation of the resulting embryo into the female body, is the most common approach in assisted reproductive technology. Since one of the important causes for male infertility is reduced sperm motility, one important consideration for in vitro fertilization is to increase sperm motility. Microswimmers are particularly suited for this task, and thus many “spermbots”, i.e., biohybrid microswimmers where the living component is a sperm cell, have been developed. Although spermbots act by delivering a type of cargo, and sometimes rely on physical manipulation, this review chooses to discuss them separately because of their highly specialized nature. Furthermore, for a more in-depth overview of this topic, interested readers are directed to two other recent review papers focused on spermbots and their potential for in vitro fertilization [92,93].

**Figure 6:**
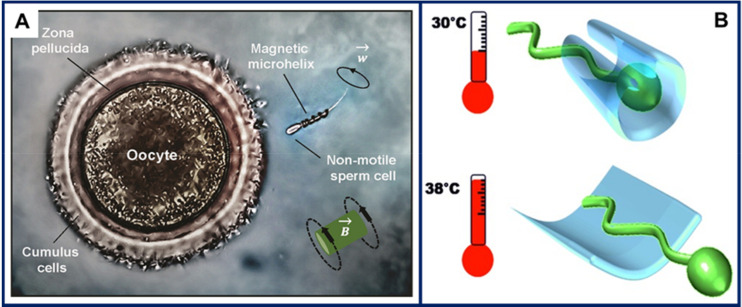
Spermbots for assisted fertilization. (**A**) An immotile sperm cell is captured by a remotely controlled magnetic helix and delivered to the oocyte for fertilization. Figure reproduced from [1], © 2015 American Chemical Society. (**B**) Thin thermoresponsive ferromagnetic multilayers roll into microtubes to help capture sperm cells and remotely control them by external magnetic fields. The polymeric spermbots are propelled by the sperm flagella. When the temperature is increased, the microtubes unfold and the sperm cell is released. Figure reproduced from [94], © 2016 WILEY-VCH Verlag GmbH & Co.

Schmidt’s group pioneered spermbots less than 10 years ago. The first spermbots used highly motile bovine sperm cells as a complementary propulsion system to the magnetic microtube component of the biohybrid microswimmer [3]. Later, the group reported spermbots with potential in assisted fertilization [1,94]. A schematic representation of the in vitro assisted fertilization concept is shown in Figure 6A: using remote magnetic actuation, a helical microswimmer is used to capture and transport a non-motile sperm cell to the oocyte, which is the target site for fertilization [1]. Capture and release of the non-motile sperm cell were based on mechanical interactions: the magnetic microhelix was guided to fit around the sperm tail and used for pushing the sperm toward the fertilization site. A more controlled approach for sperm capture and release was described soon after [94]. The magnetic microhelix spermbot component was replaced by an ultrathin thermoresponsive ferromagnetic polymeric microtube. Although this approach allowed controlled release of the sperm cell by simply increasing the temperature by a few degrees, as shown in Figure 6B, spermbot propulsion also exploited sperm cell motility.

IRONSperms are spermbots where the sperm cell component has reduced motility and the non-living component consists of magnetic elongated maghemite nanoparticles [7]. The sperm-templated biohybrids were shown to be biocompatible and responsive to both magnetic and acoustic cues. However, IRONSperms were developed for drug delivery and not for assisted fertilization, as the other spermbots discussed in this section.

In principle, one interesting potential use for spermbots is bypassing the need for in vitro fertilization by performing assisted fertilization directly in vivo. For this purpose, the microswimmers need to be biocompatible and their motion needs to be controllable in in vivo environments. One of the latest spermbot demonstrations from Striggow et al. focused on this aspect and optimized the microswimmer shape for motion in bovine oviduct fluid [95].

Whereas the incorporation of sperm cells in spermbots is a rather straightforward approach for enhancing sperm cell motility, an interesting alternative was reported by Debnath et al., who performed numerical simulations of motility transfer between different types of particles [96]. Although their model suggests that mixing sperm cells of low motility with actively-moving microswimmers could be employed for enhancing sperm cell motility, the model still needs experimental validation, as well as a practical demonstration of its advantages compared to the use of spermbots.

### 3.2. Sensing

Microswimmers can be employed for sensing by monitoring different parameters, such as motion changes, fluorescence, or electrochemical signals [54]. The said changes could be observed directly in the microswimmers, or in their liquid environment. In biomedical applications, the use of microswimmers for sensing can e.g., contribute to clinical diagnosis or complement targeted drug delivery by monitoring localized drug-induced effects. However, sensing is not specific to biomedical applications, as detection and quantification of various compounds is also of interest in e.g., environmental applications.

Microswimmers have been employed for sensing toxic chemical agents, biological agents, or in immunoassays, as recently reviewed elsewhere [54]. The first example of microswimmer sensing was motion-based heavy metal detection and came from Kagan et al. in 2009 [97]. Other motion-based examples followed, such as the platinum/polypyrrole enzyme-modified Janus nanorods that can sense the concentration of the corresponding enzymatic substrate in their environment [98], or copper/platinum concentric bimetallic microtubes able to detect lead in water [99]. Recently, simple polystyrene microspheres catapulted using optical forces in a simple mucus model were shown to exhibit different velocities and trajectories in the presence of different mucin concentrations [100].

In 2017, Campuzano et al. reviewed the use of microswimmers for (bio)sensing at the cellular level [101], and in 2018, Kim et al. discussed at length the use of microswimmers in diagnostic sensing [102]. Since then, additional noteworthy examples have emerged, as detailed below.

Local flow sensing using ferromagnetic helical microswimmers was reported by Barbot et al. [103]. A combination of simulations and experimental measurements was employed to estimate and validate the microswimmer motion before flow sensing could be achieved. Local flow sensing followed by semi-automatic algorithms that compensate for flow changes can help adjust the microswimmer control and optimize its movement, which is an important step toward user-friendly applications, or for microswimmer navigation in blood vessels.

Another approach for improving the control of autonomous swimming was reported by Yoshida and Onoe, who developed smart microswimmers able to detect and react to cues in their environment [104]. The soft spiral-shaped microswimmers consisted of a polymeric bilayer comprising both a stimuli-responsive hydrogel and a non-responsive hydrogel. The smart material component gradually responded to temperature changes by expanding or contracting, enabling microswimmer adaptation to the surrounding thermal stimuli. Stimuli-responsive materials were also employed for fabricating soft microswimmers with proteolytic degradation, which occurred at a rate depending on the matrix metalloproteinase 2 enzyme concentration [24].

**Figure 7:**
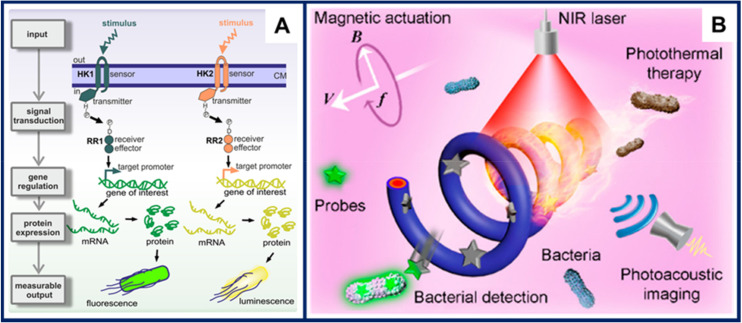
Recent examples of microswimmers with sensing capabilities. (**A**) Signal transduction pathway engineered into biohybrid microswimmers based on *Bacillus subtilis*. The presence of certain stimuli in the microswimmer environment ultimately leads to a measurable output, either fluorescence or chemiluminiscence. Figure reproduced from [105], © 2019 by the authors (Creative Commons Attribution license). (**B**) Microswimmers developed for the diagnosis and treatment of multi drug resistant *Klebsiella pneumoniae.* The microswimmers combine propulsion by magnetic actuation, bacterial detection using fluorescent markers, photoacoustic imaging, and photothermal therapy enabled by near-infrared (NIR) illumination. Figure reproduced from [106], © 2020 American Chemical Society.

Biohybrid microswimmers based on *Bacillus subtilis* and abiotic particles were reported by Sun et al. [105]. Interestingly, the bacterial components were genetically engineered to express fluorescent proteins upon encountering certain environmental stimuli. More specifically, two populations of microswimmers able to sense the antibiotic bacitracin were demonstrated. The first one expressed the mCherry red fluorescent protein, while the second expressed the green fluorescent protein (GFP). The cascade of biological processes leading from an environmental input to a measurable output, known as signal transduction pathway, is shown in Figure 7A.

Magnetic microswimmers consisting of a magnetized *Spirulina* matrix coated with polydopamine with grafted coumarin 7 molecules were employed for on/off fluorescence diagnosis [106]. In the “normal” state, i.e., in the absence of the target analyte, the characteristic green fluorescence of coumarin 7 could not be observed, due to a quenching caused by the polydopamine layer. Upon encountering the target *Klebsiella pneumoniae* bacteria, coumarin 7 detached from the polydopamine layer and attached to the bacteria via strong electrostatic interactions, leading to the appearance of a green fluorescence signal in the sample. After confirming the presence of the target analyte, the microswimmers were employed for near infrared-activated photothermal therapy, both in vitro and in vivo in mouse subcutaneous *Klebsiella pneumonia* infections. (Figure 7B).

Catalytic microswimmers displaying chemiluminescence based on an interfacial redox process were recently developed by Salinas et al. [107] The microswimmers were Janus particles with a cathodic magnesium region and an anodic water-soluble electrophoretic paint with negatively charged carboxyl groups. The microswimmers were tested on the interface of a H_2_O/ACN (1/1) solution containing 1 mM Ru(bpy)_3_(PF_6_)_2_, 20 mM K_2_S_2_O_8_, and 20 mM H_2_SO_4_. Whereas mechanical motion was achieved based on magnesium oxidation leading to bubble propulsion; the chemiluminescence appeared due to the presence of Ru(bpy)_3_^2+^ and S_2_O_8_^2−^. Although sensing was not demonstrated in this paper, the presented microswimmers could in principle be used for sensing chemiluminiscence quenchers.

Another interesting demonstration came from Dasgupta et al., who reported microswimmers with the ability to sense local physicochemical heterogeneities in tumor microenvironments [108]. In their paper, silica-based helical microswimmers containing ferromagnetic components injected in a model breast tumor microenvironment tended to adhere in the extracellular matrix in the vicinity of cancer cells, whereas the same behavior was not observed near healthy cells. Surface coating of the microswimmers with 1H,2H,2H-perfluorooctyltriethoxysilane, a chemical compound able to “shield” from charged environments, significantly reduced the microswimmer adhesion near cancer cells. This provided insight into the cancer cell cue leading to adhesion, namely a charge-based mechanism.

### 3.3. Microswimmer-Mediated Object Manipulation

The precise control potential of certain types of microswimmers is an excellent opportunity for object manipulation, including for applications in biomedical studies. For example, targeted tissue penetration and deformation was demonstrated using perfluorocarbon-loaded microbullets [109]. The perfluorocarbon emulsion used as “explosive” was loaded into the microbullets by electrostatic binding on a thiolated cysteamine monolayer adsorbed onto the microbullet interior gold layer. The bullet-like behavior was activated by ultrasound stimulation, which triggered rapid vaporization of the perfluorocarbon droplets leading in turn to energy transfer.

Indirect manipulation of colloidal particles was achieved with the aid of light-controlled thermoplasmonic microswimmers [110]. The 3D printed microswimmers amenable to optical manipulation included a gold-coated disk. When illuminated, the gold-coated disk rapidly heated due to thermoplasmonic effects, inducing a natural convection flow in its surroundings, which in turn caused nearby colloidal particles to move in a toroidal pattern.

Manipulation of individual silica particles and of HeLa cells using acoustically-powered microswimmers was recently demonstrated [111]. The microswimmers consisted of microcapsules fabricated by 2PP 3D printing coated with nickel and gold. Air bubbles were trapped into the microcapsules by incubation in trichloro(1H,1H,2H,2H-perfluorooctyl) silane vapor, which caused the formation of a hydrophobic monolayer. The microswimmers had dual actuation, magnetic and acoustic, and demonstrated a precision for in-plane particle manipulation similar to that of optical tweezers and potential for 3D manipulation.

Cogwheel-shaped microswimmers with precise optoelectronic tweezers control were fabricated using standard SU-8 photolithography and applied for the indirect micromanipulation of different types of objects, including microparticles and cells [112]. The use of optoelectronic tweezers enabled simultaneous actuation of several microswimmers.

### 3.4. Imaging

Although the imaging of biomedical microswimmers in complex samples and ultimately in the human body is an important topic [113,114], this section refers instead to the applications of microswimmers as agents for improved imaging.

**Figure 8:**
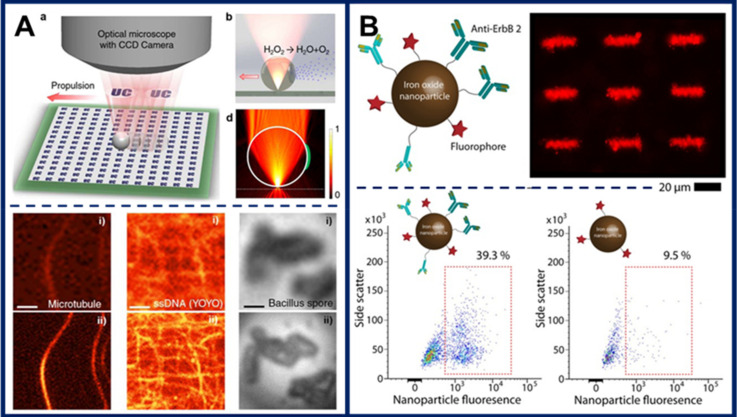
(**A**) Microswimmer-enabled optical nanoscopy. (Top) Schematics showing the operating principle for microswimmer-enhanced imaging. (Bottom) Imaging of fluorescent and biological samples with and without microswimmer-enabled optical nanoscopy. The images acquired with the aid of microswimmers have significantly better resolution. Figure reproduced from [115], © 2016 American Chemical Society. (**B**) Targeted cell labeling using magnetic nanoparticles released from collapsed microswimmers. (Top) Design of superparamagnetic iron oxide nanoparticles functionalized with a fluorophore and an antibody for targeted labeling of breast cancer cells and epifluorescence image of microswimmers embedded with the nanoparticles. (Bottom) Labeling of the breast cancer cells shows much higher efficiency for nanoparticles modified with the antibody. Figure reproduced from [24], © 2019 American Chemical Society (Creative Commons Attribution license).

The use of microswimmers for optical nanoscopy enabling sub-diffraction resolution was demonstrated by Li et al. [115]. For this purpose, they fabricated Janus microswimmers made of high refractive index polystyrene or of titania microspheres partially coated with a thin titanium/nickel/platinum metallic trilayer. In the presence of hydrogen peroxide fuel, the asymmetric microswimmers swim autonomously, powered by platinum-catalyzed fuel decomposition. This motion is complemented by magnetic actuation enabled by the nickel component. At the microscale, each microswimmer acts as a magnifying lens, whereas motion control enables precise positioning near the features of interest and scanning of relatively large areas of a sample in a short amount of time. After suitable image processing, features as small as 20 nm could be resolved, and high-resolution imaging of neuron axons, microtubulin, *Bacillus* spores, and DNA nanotubes was demonstrated (Figure 8A).

Microswimmer-enabled fluorescent labeling represents a different concept that can be applied to improving imaging. In this case, the action mechanism is cargo transport, but the application lies in imaging, as well as in drug delivery. Ceylan et al. fabricated hydrogel microswimmers containing superparamagnetic components by 2PP 3D printing [24]. For targeted cell labeling, magnetic nanoparticles tagged with both antibodies and fluorophores were embedded into the soft microswimmers (Figure 8B). Upon matrix metalloproteinase-induced microswimmer degradation, fluorescent labeling of the target breast cancer cells was demonstrated. This represents an interesting approach with potential for “microsurgeries”, where a single population of microswimmers could be employed as a drug delivery system, as well as for labelling the target cells for subsequent evaluation of the success of the procedure.

Another noteworthy example came from Hosseini et al., who reported *Spirulina*–bismuth biohybrids for enhanced computed tomography imaging [116]. Although it can be argued that these biohybrids do not fit the profile of microswimmers well, because they are neither self-propelled nor externally controlled, their fabrication strategy and in vivo application are definitely relevant. *Spirulina platensis* microalgae were employed as biotemplates for the one-pot synthesis of microrods coated with a high content of bismuth nanoparticles, which could be converted to hollow microrods by calcination. The X-ray absorption of the biohybrids was compared to that of traditional contrast agents and showed slightly better contrast at the same concentration. In vivo contrast tomography imaging was done in rats after using both oral gavage and intravenous injection for administration.

### 3.5. Microsurgery

Laser-controlled microswimmers for intraocular surgery were recently reported [14]. The microswimmer design was inspired from nature and aimed to mimic shrimp locomotion. The microswimmers were fabricated by focused ion beam milling of nitinol, which is a nickel/titanium shape memory alloy. By using a combination of direct optical trapping and optothermal forces, a microswimmer could be precisely controlled and exhibited a shrimp-like crawling motion. Whereas the authors suggest the use of such microswimmers for intraocular surgery, biocompatibility and testing and additional safety demonstrations are required before this could be implemented in practice.

Earlier this year, Vyskočil et al. reported microrobotic scalpels which could enter the cytoplasm of cancer cells and remove a piece of the cytosol without destroying the cell membrane [117]. The microscalpels were made of alternating gold, silver and nickel segments and were controlled by applying a transversal magnetic field. Upon actuation, the microscalpels exhibit a tumbling motion and act as surface walkers rather than microswimmers.

## 4. From Laboratory Studies to In Vivo Applications and Commercialization

Early microswimmer studies explored various fabrication and actuation techniques. Although improvements are still being made in these areas, most recent studies focus instead on demonstrating the usefulness of microswimmers for target applications. In the case of biomedical applications, there is an ever-growing interest to shift from laboratory studies towards clinical use, which is why many in vitro and in vivo pre-clinical studies are being conducted worldwide. To the best of our knowledge, there are no clinically approved microswimmers and no ongoing clinical trials involving microswimmers. However, several pre-clinical animal studies have shown minimal adverse effects [19].

**Figure 9:**
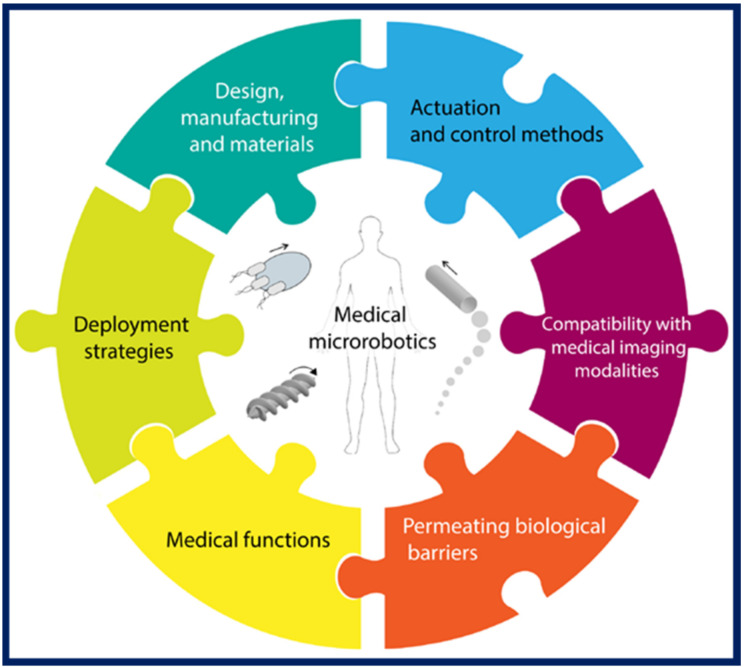
Essential considerations for biomedical microswimmers for in vivo applications, according to Ceylan et al. [119]. Figure reproduced from [119], © 2019 by the authors (Creative Commons Attribution license).

Currently, many of the most recent microswimmer studies focus on demonstrating different elements required for in vivo use. When it comes to microswimmer applications in the human body, it is already well known that many prerequisites need to be met before clinical translation. Some of these are rather obvious, such as the critical need for biocompatibility and long-term biosafety, while others require a more in-depth consideration of the situation, such as the microswimmers’ ability to permeate biological barriers, or their compatibility with in vivo imaging techniques. The essential considerations for successful translation of microswimmers to in vivo biomedical applications were recently discussed by several groups [68,118,119,120]. Figure 9 shows a schematic representation of these essential considerations, as described by Ceylan et al. [119].

Significant efforts have recently been put into producing biocompatible microswimmers, and important progress has already been made, leading to the fact that many of the latest microswimmers are made using biocompatible materials [121] and/or biocompatible propulsion mechanisms [122]. However, the long-term biocompatibility and biosafety of the entire microswimmer system, including any potential external actuator, still needs to be evaluated before clinical translation could become a reality. On the other hand, microswimmer retrieval is an important part of the long-term biosafety, as accumulation of microstructures in the human body is definitely not desirable. Whereas for some applications, microswimmer elimination through urine or feces might be achievable, others require deep tissue penetration or even intracellular access, in which case the natural elimination pathways are less likely. Thus, as many examples covered by this review show, the latest trend involves the use of biodegradable microswimmers, in which case retrieval becomes unnecessary. Nevertheless, even for biodegradable microswimmers, long-term studies need to confirm their biocompatibility and biosafety before deployment in humans can become a reality. This represents a significant bottleneck, because obtaining approval from the various regulatory bodies, such as the Food and Drug Administration (FDA) in the USA or the European Medicine Agency (EMA), is notorious for being laborious and expensive, and for having a very low success rate.

Technical challenges, especially regarding mass-production, are particularly important when it comes to the microswimmer commercialization potential. Many of the technologies currently employed in the fabrication of various types of microswimmers have relatively low throughput, whereas more established mass-production technologies do not offer compatible solutions for processing the required materials with sufficient resolution. Furthermore, some of the most promising microswimmer examples are based on smart materials with encoded functionality, which are often challenging to produce on a large scale. Altogether, these issues make the fabrication of microswimmers a costly process in terms of both money and time. To compensate for this, microswimmers would need to be truly unparalleled solutions to certain biomedical challenges before becoming cost-effective for commercialization purposes.

The many recent examples included in this review show that researchers are exploring various fundamentally different strategies when it comes to the design, fabrication, functionalization and actuation of microswimmers for biomedical application. All these different strategies have their characteristic advantages and disadvantages, which can manifest with regard to many aspects that can influence their real-world potential, including e.g., their success for the target application, biocompatibility, in vivo behavior and tracking possibilities, potential for mass-production, or operational costs. Currently, given the relatively recent emergence of the field and the lack of examples approved for real-world use, there is no established “path to success” for microswimmers. Furthermore, we believe that microswimmer solutions will not necessarily be universal, meaning that different target applications would benefit more from applying one combination of strategies over another. Consequently, the aim of this review is not to favor certain strategies, but rather to provide an overview of what has been reported to date, as well as hinting at important aspects to consider when developing future microswimmers.

## 5. Conclusions and Outlook

Various types of microswimmers have shown excellent promise at laboratory level for a number of different biomedical applications. Among these, cargo transport and drug delivery are likely the most explored, while other relevant applications are in vitro insemination, sensing, indirect object manipulation, imaging, and microsurgery. In recent years, microswimmer research has been focused on various aspects necessary for clinical translation, and pre-clinical studies have so far shown promising results. Although the concept of personalized medicine dates back to ancient times, it has grown tremendously in recent years, supported by developments in various areas of technology. Microswimmers provide interesting perspectives for many biomedical applications, and would likely foster unique solutions for personalized medicine, provided that clinical trials prove successful and deployment of microswimmers into the human body becomes a widely accepted medical procedure.

The envisioned benefits of using microswimmers in vivo for treating various human diseases and conditions are beyond doubt. However, there are many laboratory studies of clinical relevance for which microswimmers should be particularly suitable. Although the use of microswimmers in vivo is a commendable long-term goal, in vitro or ex vivo biomedical applications should be achievable in a much narrower timeframe. Therefore, we believe that the huge potential of microswimmers for biomedical applications at a laboratory level should not be forgotten in the “race” to reach the human body. The precise control of microswimmer motion and cargo delivery could help foster our fundamental understanding of e.g., localized vs. generalized drug effects, side-effects, etc. For example, consider a microswimmer-assisted controlled administration of a drug or drug delivery system to a few selected cells in a monolayer cell culture. Monitoring and comparing the response of treated cells, as well as that of other cells from the same monolayer that were not exposed to the drug, could provide a much more detailed understanding of the drug delivery process than when using separate cell populations for the study. A similar approach could be used for investigating the dose response for a therapeutic agent. In this case, microswimmers could deliver different amounts of drug to different areas of the same cell culture, and the cell response to the different dosages could be analyzed independently from other factors. Another potential laboratory application for microswimmers is in diagnostics, where many of the various tests are typically performed ex vivo. Tailored microswimmers could help detect extremely low concentrations of a target analyte by “probing” the sample, i.e., physically moving in the biological fluid or tissue. Alternatively, microswimmers enabling improved imaging might provide an interesting solution for diagnostic purposes. Harvesting these relatively low-hanging fruits might provide the necessary boost to enable microswimmers to move beyond the boundaries of research laboratories.

Immense efforts have already been dedicated to the development of microswimmers for different tasks, of which biomedical applications are the most widespread. Before becoming well-established and accepted solutions to biomedical issues, microswimmers need to comply with strict requirements from regulatory bodies, although all signs point towards a brilliant future for cleverly engineered microswimmers, capturing the public’s attention requires demonstrating interesting applications in the real world. Furthermore, prolonged public enthusiasm would likely require the microswimmers to be produced on a large scale at a cost allowing for profitable commercialization Thus, after a demanding yet relatively smooth swimming course so far, microswimmers need to brace for encountering significant challenges in the near future.

Despite being aware of the challenges ahead, we would like to highlight once again the hitherto extremely rapid development of microswimmers. We believe that the huge interest and intellectual involvement in the field are likely to contribute to suitable solutions to translational challenges, and it is just a matter of time until microswimmers can take their rightful place in diagnostic labs, clinics and hospitals.
